# Genetic Analysis of *MMP-2* and *MMP-3* Polymorphisms Reveals the Association of *MMP-3* rs522616 with Susceptibility to Persistent Apical Periodontitis

**DOI:** 10.3390/genes16070758

**Published:** 2025-06-28

**Authors:** Tulio L. Olano-Dextre, José F. Mateo-Castillo, Celso K. Nishiyama, Carlos F. Santos, Gustavo P. Garlet, Renato M. Silva, Ariadne Letra, Lucimara Teixeira das Neves

**Affiliations:** 1 Post-Graduation Program in Rehabilitation Sciences, Hospital for the Rehabilitation of Craniofacial Anomalies, University of São Paulo, Bauru 17012-900, SP, Brazil; olanotulio@hotmail.com (T.L.O.-D.); cmfj88@hotmail.com (J.F.M.-C.);; 2 Department of Endodontics, Hospital for Rehabilitation of Craniofacial Anomalies, University of São Paulo, Bauru 17012-901, SP, Brazil; kenjiusp@uol.com.br; 3 Department of Biological Sciences, Bauru Dental School, University of São Paulo, Bauru 17012-901, SP, Brazil; garletgp@usp.br; 4 Department of Endodontics, University of Pittsburgh School of Dental Medicine, Pittsburgh, PA 15219, USA; renato.silva@pitt.edu (R.M.S.); ariadneletra@pitt.edu (A.L.); 5 Department of Oral and Craniofacial Sciences, Center for Craniofacial and Dental Genetics, University of Pittsburgh School of Dental Medicine, Pittsburgh, PA 15219, USA

**Keywords:** persistent apical periodontitis, matrix metalloproteinase, single nucleotide polymorphism, genetic predisposition, endodontics

## Abstract

**Objective**: The aim of the present study was to investigate the association of polymorphic variants in matrix metalloproteinase-2 (*MMP-2*) and matrix metalloproteinase-3 (*MMP-3*) genes and the occurrence of persistent apical periodontitis (PAP). **Methods**: DNA samples from 180 individuals were recruited and divided into two groups: Case group, 79 subjects with a history of PAP; control group, 101 healthy subjects. Five single nucleotide polymorphisms (SNPs) were selected for genotyping: rs243865, rs2285053, and rs2287074 in the *MMP-2* gene, and rs679620 and rs522616 in the *MMP-3* gene. The chi-square test or Exact Fisher (*p* < 0.05) and Odds Ratio (CI = 95%) were used to compare the frequencies of genotypes and alleles between the two groups. **Results**: A positive association was found for the *MMP-3* rs522616 AG genotype (*p* = 0.025), the AG + GG genotypes (*p* = 0.015), and the G allele (*p* = 0.016) with PAP. **Conclusions**: The *MMP-3* variant rs522616 was associated with PAP. Similar studies are needed analyzing other genes involved in extracellular matrix dynamics under inflammatory conditions to clarify the role of the genetic factors of PAP.

## 1. Introduction

Apical periodontitis is an inflammatory response of the periapical tissues, usually associated with bacterial infections resulting from alterations in the root canal, such as necrosis or pulp infection. The interaction between the root microbiome and host immuno-inflammatory factors plays a crucial role in the pathogenesis of this condition. In this context, non-surgical intervention through root canal treatment (RCT) is necessary. RCT involves the removal of dental pulp compromised by necrosis or inflammation, followed by chemical and mechanical disinfection of the root canal system and subsequent sealing with biocompatible materials. The aim of RCT is to clean, conform, disinfect, and seal the three-dimensional root canal system to eliminate or significantly reduce microorganisms in order to eliminate the trigger of a local and destructive chronic inflammatory immune response and, consequently, allow the repair of the periapical region. However, failure may occur, and thus persistence of infection, the persistent local response, and the consequent periapical lesion [1,2,3].

Persistent apical periodontitis (PAP) is defined as a chronic inflammatory periapical lesion related to a non-vital tooth in which there is no reestablishment of the periapical tissues after root canal treatment (RCT) [2,4,5,6,7]. PAP is a multifactorial condition, related not only to factors such as microbiological and physical–mechanical factors, but also especially intimately related to the host response in the individual’s ability to repair [3,6]. PAP may present symptoms such as persistent or recurrent pain, often triggered by pressure or chewing, sensitivity to percussion or palpation, the presence of fistula or purulent drainage, and, in advanced cases, tooth mobility due to degradation of the bone support. However, it often develops without symptomatology and, for this reason, imaging plays an important role in its detection and diagnosis [3,8,9]. Although some of these lesions are asymptomatic, they constitute an important risk to the individual’s health, since this type of lesion has been associated with the occurrence of systemic diseases [10,11,12,13].

While the persistence of the microbial insult is one factor for PAP, the nature and extent of the host immune response to this aggressive agent is ultimately responsible for determination of the lesions’ outcome [13,14]. Indeed, the immune response can be modulated in different ways, depending on some systemic conditions of the individual and also on their genetic profile, often associated with polymorphic variants (single nucleotide polymorphisms—SNPs) [15,16]. SNPs are the most common type of genetic variation in the human genome and may affect gene expression [17]. These variants can influence the inflammatory and immune responses. In the context of endodontic treatment, SNPs have been linked to variable responses to root canal treatment (RCT), depending on the patient’s genetic profile. This genetic variability may also contribute to the development of certain diseases, such as PAP [2,6,14,15,16,18,19,20].

The host’s local immune response to microbial factors triggers a cascade of events involved in healing, including vascular and cellular inflammatory events; cell migration, proliferation and differentiation; angiogenesis; matrix deposition; and remodeling of compromised bone tissue [21]. An imbalance in this cascade of pro- and anti-inflammatory events that have the ultimate goal of promoting the healing of the lesions may lead to tissue destruction [22]. In this context, periapical lesions are a consequence of this local immune imbalance in the periapical environment, resulting in the destruction of the soft and mineralized tissues surrounding the apex [21,23].

Among the enzymes responsible for the degradation of the pericellular substrate or extracellular matrix are the matrix metalloproteinases (*MMPs*) [7,24]. *MMPs* comprise an important family of metal-dependent endopeptidases, initially secreted as pro-enzymes and requiring extracellular activation, and regulated by endogenously secreted inhibitors [25,26]. The persistence of microorganisms after root canal treatment has been associated with the presence of severe tissue disorganization and the exacerbated local expression of *MMPs* in the periapical areas, being related to their various functions in the bone tissue such as remodeling and the immune response [24,25,27]. Studies observing a significant increase in the expression levels of *MMP-1*, *MMP-2*, *MMP-3*, *MMP-7*, *MMP-9*, and *TIMP 1* could be related to an individual predisposition to apical periodontitis (AP) [24,26,28,29,30].

Building on the evidence that links matrix metalloproteinase (MMP) expression and genetic variations to apical periodontitis (AP), attention has turned to its persistent forms, such as persistent apical periodontitis (PAP). Persistent apical periodontitis (PAP) is a chronic inflammatory condition that can occur as a complication of endodontic treatment, characterized by a failure to resolve the infectious or inflammatory process in the periapical tissues. This condition not only represents a challenge in clinical dental practice but also has broader implications for public health, considering its prevalence and impact on patients’ quality of life [2,3,6,7]. Recent studies have demonstrated an association between single nucleotide polymorphisms (SNPs) in the matrix metalloproteinase (MMP) genes and increased susceptibility to apical periodontitis (AP), reinforcing the role of these enzymes in extracellular matrix remodeling, inflammation control, and the progression of chronic diseases [26,28]. However, to date, no study has evaluated the relationship between genetic variants of the *MMP-2* and *MMP-3* genes as possible host susceptibility factors for PAP. This scientific gap limits more effective preventive interventions and personalized approaches in at-risk populations. In this context, the aim of the present study was to investigate the association of the SNPs rs243865, rs2285053, and rs2287074 in the *MMP-2* gene, as well as rs679620 and rs522616 in the *MMP-3* gene, with the occurrence of persistent apical periodontitis (PAP). This study sought to advance the understanding of the genetic factors underlying PAP and to provide evidence that may support the development of improved strategies for the diagnosis, prevention, and management of this condition.

## 2. Materials and Methods

### 2.1. Ethical Approval

According to the principles of the Declaration of Helsinki, this study was approved by the Committee of Ethics in Human Research of the Hospital of Rehabilitation of Craniofacial Anomalies, University of São Paulo (HRAC/USP) (approval CAAE number 25300713.4.0000.5441). Informed written consent was obtained with an assent document signed by all subjects.

### 2.2. Study Participants

**Figure 1:**
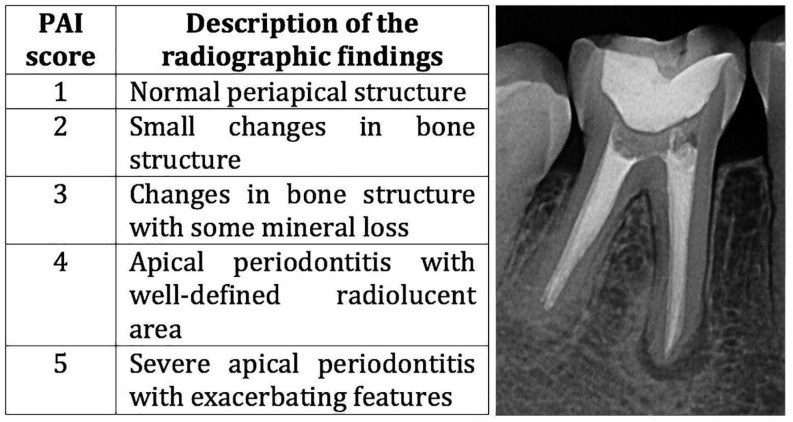
Description of the PAI score and an example of a PAI score of 4 of a subject from the case group.

Initially, the post-treatment periapical radiographs of 2888 subjects from the southeastern region of Brazil who received RCT at the Endodontic Clinic of Bauru School of Dentistry and Hospital of Rehabilitation of Craniofacial Anomalies, University of São Paulo, were analyzed. Regarding the inclusion/exclusion criteria for the case group, we selected cases that presented teeth with definitive restoration and endodontic treatment with persistent periapical lesions after endodontic treatment. In these individuals, the PAP should be equal to or greater in size than the lesion present in the periapical radiograph at the end of the endodontic treatment, with persistence of the lesion characterizing PAP. Radiographs at the end of the endodontic treatment and those performed in the follow up period after the root canal treatment (RCT) were evaluated according to the periapical index (PAI), which defines scores from 1 to 5 for present lesions [31] (Figure 1).

The detailed criteria for assigning the scores are defined as follows (Figure 1): as Score of 1 radiographically presents a standard periapical structure with the periodontal ligament space and an intact lamina dura; a score of 2 presents minor radiographic changes in the bone structure surrounding the apex; a score of 3 presents radiographic changes in the bone structure with loss of mineral tissue; a score of 4 presents radiographic changes in the periapical region with changes in the periodontal ligament space, loss of integrity of the lamina dura with changes in the bone structure, and well-defined radiolucent areas; and a score of 5 presents radiographic changes in the periapical region with severe apical periodontitis with areas of exacerbation.

For the present study, only subjects presenting scores of 4 or 5 with one or more previously treated teeth were selected for follow-up after 1 year or more. Individuals with periapical radiographs with scores less than 4 or 5 or who were poorly processed were excluded from the study. Prior to the analysis of the PAI index, an examiner was previously trained by an experienced radiologist. After training, the examiner was calibrated in a pilot study with 40 periapical radiographs that were randomly selected for the pilot study). The 40 periapical radiographs were evaluated twice within 15 days. The results were tabulated in the Microsoft Excel program and submitted to the Kappa test. The intra-examiner agreement in the Kappa test was 0.79, which is classified as substantial according to the parameters established by Landis and Koch [32].

For the control group, healthy individuals, whose current condition did not require dental treatment, were selected. Therefore, this case–control study included 180 subjects (79 cases and 101 controls) and was conducted using a convenience sample, selected according to the accessibility and availability of participants for the research.

### 2.3. DNA Extraction

Saliva samples were collected from all subjects included at the time of the follow-up visit. Saliva was collected by expectorating in a 15 mL propylene tube. The samples were stored at −20 °C until the beginning of the DNA extraction step. For the analysis of allelic discrimination, genomic DNA was extracted from buccal cells isolated from the saliva. Genomic DNA was performed with the QIAamp DNA extraction protocol kit (Qiagen, Valencia, CA, USA) following the manufacturer’s instructions. Shortly after the saliva samples were thawed entirely, each tube containing 1.5 mL was well homogenized in a vortex mixer. The following were pipetted into a 2 mL microtube: 20 µL of proteinase K, 200 µL of well-homogenized saliva, and 200 µL of the buffer AL. Each sample was vortexed for 15 s and then placed in a water bath for 10 min at 56 °C. They were then quickly centrifuged to remove drops from the cap; 200 µL of absolute alcohol was added, vortexed for 15 s, and quickly centrifuged. The contents were transferred to the QIAamp column and centrifuged at 15,000 rpm for one minute at room temperature. The column was transferred to a new microtube, and 500 µL of the buffer AW1 was added and centrifuged for one minute at room temperature. The column was transferred to a new microtube, and 500 µL of the buffer AW2 was added and centrifuged at 15,000 rpm for 3 min at room temperature. Then, the column was dry centrifuged in another new microtube at 15,000 rpm for one minute at room temperature. The column was placed in a 1.5 mL microtube, and 50 µL of the buffer AE was added and centrifuged at 15,000 rpm for one minute at room temperature. The DNA samples were frozen at −20 °C until DNA quantification in a spectrophotometer.

The amount and purity of the DNA were determined by a spectrophotometer (NanoDrop™ 1000, Thermo Fisher Scientific, Wilmington, DE, USA). DNA samples presenting an A260 nm/A280 nm ratio lower than 1.8 were subjected to the extraction process again to ensure DNA purity before the genotyping experiments. The initial amount of DNA for genotyping was standardized at 50 ng/μL, so all samples were diluted to this final concentration.

### 2.4. Selection of Candidate Genes and SNPs

Five SNPs were selected for the genetic evaluation, three of them in the *MMP-2* gene, namely rs243865 (Life Technologies^®^ Carlsbad, CA, USA, catalog C___3225943_10), rs2285053 (Life Technologies^®^ catalog C__26734093_20), and rs2287074 (Life Technologies^®^ catalog C___3225956_10), and two in the *MMP-3* gene: rs679620 (Life Technologies^®^ catalog C___3047717_1_) and rs522616 (Life Technologies^®^ catalog C___3047714_10). These polymorphisms were chosen according to the studies of Menezes-Silva et al. (2012) [28]. The features of the genetic polymorphisms are presented in Table 1.

### 2.5. Genotyping

Genotyping was performed by real-time polymerase chain reaction (PCR) using the TaqMan method. The reactions were performed on a Viia 7 sequence detection system (Applied Biosystems, Foster City, CA, USA) using validated TaqMan SNP genotyping assays (Applied Biosystems, Foster City, CA, USA) following the manufacturer’s instructions. The experiments were performed in duplicate on 384-well plates. A duplicate negative control (non-template control, NTC) was included in this PCR reaction plate to check for possible contamination. For this purpose, the reactions were performed containing all reagents in the mixture (primers, probes, buffer, enzymes, and nuclease-free water) but without the addition of the target DNA or RNA. The absence of an amplification curve in the negative control confirmed the specificity and purity of the reagents used in the experiment. For each reaction, a final product of 5 μL containing 2 μL of the DNA sample at 50 ng/μL, 2.5 μL of TaqMan Genotyping Master Mix (Applied Biosystems, Foster City, CA, USA), 0.125 μL of each TaqMan assay (Applied Biosystems, Foster City, CA, USA), and 0.375 μL ultrapure water qsp. The thermal cycling was performed by starting with a hold cycle of 95C for 10 min, followed by 40 amplification cycles of 92C for 15 s and 60C for 1 min. The cycle threshold (Ct) corresponds to the number of cycles required for the fluorescent signal to exceed the established threshold. In a real-time PCR assay, a positive reaction is identified by the progressive increase in the fluorescent signal, with Ct values having an inverse relationship with the concentration of the target nucleic acid present in the sample. Genotypes were called using EDS software (version 1.1, Applied Biosystems, Foster City, CA, USA).

All analyses were performed at the Pharmacology and Genetics Laboratory of the Bauru Dental School, University of São Paulo. The probes and the master mix were from Applied Biosystems (Foster City, CA, USA). All researchers who carried out the tests in the laboratory were unaware of the sample group.

### 2.6. Statistical Analysis

SigmaPlot for Windows 12.0 software was used for the data analysis. The Chi-square test (Χ^2^) or Fisher’s exact test and odds ratios with confidence interval (CI) = 95% were used to compare genotype and allele frequencies between the groups. Values of *p* < 0.05 were considered statistically significant. The Hardy–Weinberg equilibrium was evaluated using the Chi-square test within each polymorphism (https://geneswellness.com/chi-square-hardy-weinberg-equilibrium-calculator/ accessed 24 June 2025).

A power analysis was performed using G*Power 3.1.9.7, considering the allele prevalence difference between the groups of 0.15 and an error probability of 0.05, which indicated that our sample size of 64 case subjects and 86 control subjects would present a power of 0.80.

## 3. Results

The case group was composed of 79 subjects (33 males and 46 females with an average age of 34.9 ± 15 SD), and the control group was composed of 101 subjects (28 males and 73 females with an average age of 24.7 ± 4 SD). These results reflect the approach of a convenience sample based on strict eligibility criteria and participants’ accessibility and availability for the research.

The overall genotyping success rate was 100%. Genotype distributions were in Hardy–Weinberg equilibrium. Table 2 shows the genotype and allele distributions between the groups. The results of the case vs. control groups were compared for each of the SNPs. For the SNPs rs243865, rs2285053, and rs2287074 in the *MMP-2* gene, and for the SNP rs679620 in the *MMP-3* gene, there was no statistically significant difference between the groups. For the SNP rs522616 in the *MMP-3* gene, there was a statistically significant difference associated with the AG genotype (*p* = 0.025) and the AG + GG genotypes (*p* = 0.015) with PAP (Table 2). Subjects who carry the G allele had a higher risk (*p* = 0.016; OR = 1.84; 95% confidence interval [CI], 1.11–3.02).

## 4. Discussion

The intricate interplay between genetic variation and phenotypic expression continues to be a cornerstone of contemporary biological research. In recent years, numerous studies have explored the role of genetic polymorphisms in tissue repair processes following apical periodontitis associated with root canal treatment (RCT) [2,6,18,19,20]. These investigations have increasingly emphasized the association between the host’s genetic profile and the complex molecular mechanisms driving inflammation and bone metabolism after RCT. Among the diverse genes analyzed, the MMP family have attracted particular interest due to its pivotal role in extracellular matrix remodeling and modulation of the inflammatory response [5]. This evidence highlights the critical role of genetic factors in post-treatment outcomes, underscoring the need for integrative approaches to fully understand host–pathogen interactions in endodontic pathology [3].

Some SNPs in the *MMP-2* and *MMP-3* genes have been studied, testing the hypothesis that these SNPs could be associated with increased susceptibility to apical periodontitis (AP), thereby contributing to different stages of the inflammatory process, bone remodeling, and tissue regeneration [26,28]. However, it has not yet been determined whether these polymorphisms in these genes may influence susceptibility to the occurrence of persistent apical periodontitis (PAP).

In the present study, we hypothesized that SNPs in *MMP-2* and *MMP-3* genes can influence the individual response to the repair of the periapical lesion after RCT, leading to PAP (with a PAI score of 4 or 5). The *MMP-2* and *MMP-3* genes are involved with different functions in extracellular matrix remodeling signaling pathways in numerous mechanisms related to tissue injury, and participate in the process of periapical tissue degradation which occurs in PAP [28,30]. *MMP2* encodes a gelatinase containing an active site that binds to the denatured collagen of Type IV and V collagen and elastin, while the *MMP-3* product is an enzyme that degrades Collagens III, IV, IX, and X; laminin; fibronectin; and cartilage proteoglycans [33].

The present study showed for the first time that the *MMP-3* variant rs522616 was associated with a risk of PAP. Significant associations were observed for the AG genotype and AG + GG genotypes with the occurrence of PAP. Likewise, a significant association was observed for the G allele frequency in cases with PAP when compared with the controls. In contrast, in a study conducted by Menezes-Silva et al. [28], no association was found between the rs522616 variant with an AP phenotype, whereas another *MMP-3* variant (rs679620) was significantly associated with AP. In the present study, however, no statistically significant association was found between PAP and rs679620. Collectively, these observations show that *MMP-3* is likely a plausible candidate gene for AP and PAP; however, different polymorphisms may contribute to each condition. As part of a gene family involved in bone remodeling, the *MMP-3* gene interacts synergistically with other genes in the signaling cascade for the remodeling process. In this sense, possible transcriptional changes associated with *MMP-3* rs522616 could be related to downregulation of *MMP-3* in the presence of the G allele, causing interference in the background of other genes involved in the remodeling of the bone tissue, thus leading to an impair in the apical tissue regeneration process associated with PAP. Furthermore, a study by Letra et al. [34] assessed the functional effect of *MMP-3* SNP rs522616 and found that this variant presents differential and allele-specific effects on transcription, with the A allele presenting higher promoter activity. Similarly, in another study, the presence of the allele A also increased the activity of *MMP-3* [17]. In addition, SNP rs522616 located in the gene promoter may include a binding site for transcriptional factors, and the presence of either allele (A or G) may alter the binding affinity for these factors [17].

In relation to the *MMP-2* SNPs studied, rs243865, rs2285053, and rs2287074, no associations were found with PAP. These findings corroborate those found by Menezes-Silva et al. [28]. Although *MMP-2* participates in the bone remodeling process, with significant expression in apical periodontitis in the context of expression of other *MMPs*, the polymorphic variants investigated in this study were not associated with the onset or persistence of the periapical lesion. One possible explanation for this result may be that the polymorphisms chosen in the *MMP-2* gene play a modest or even inexpressive role in controlling *MMP-2*’s expression in this process of persistence of the lesion. Also, in this cascade of molecular interactions, other factors may be more important than the SNP itself in the regulation of *MMP-2*. In this case, there could even be increased expression of *MMP-2*, but this expression would be induced by factors other than the presence of these polymorphisms.

Finally, it is worth noting that PAP is a multifactorial condition, and in the present study, we focused the analysis only on SNPs in the *MMP-2* and *MMP-3* genes. Therefore, as a recommendation for future studies, it would be interesting to include other genetic markers related to inflammation or the immune response (e.g., IL-1, TNF-α, TIMPs) that would strengthen the predictive value of the genetic model.

## 5. Conclusions

In summary, a positive association was found between *MMP-3* rs522616 and PAP in this Brazilian population. This polymorphism perhaps may be considered one of the molecular markers for the prognosis of endodontic treatment, with a modest contribution to a polygenic model involved in the host response to bacterial and inflammatory stimuli. Similar investigations analyzing other genes and with larger samples, including other geographic settings, are needed to clarify the role of the genetic predisposition to the occurrence of PAP. This knowledge could provide insights into the pathogenesis of the condition, allowing us to define risk groups for PAP’s development and proposing new alternative treatment procedures to avoid the installation of PAP and its consequences of apical bone loss.

It should be stressed that studies on a larger sample, including other geographic settings, are needed.

## Figures and Tables

**Table 1:** Characteristics of the *MMP-2* and *MMP-3* genes investigated in the study.

Gene	SNP ID	Chromosome	Functional Consequence	AA *	PA ^+^
***MMP-2***	rs243865	Chr 16:55477894	5′ UTR	C	T
	rs2285053	Chr 16:55478465	5′ UTR	C	T
	rs2287074	Chr 16:55493201	Synonymous	G	A
***MMP-3***	rs679620	Chr 11:102842889	Missense	G	A
	rs522616	Chr 11:102844317	5′ UTR	A	G

* AA, ancestral allele; ^+^ PA, polymorphic allele.

**Table 2:** Results of case–control comparisons for the associated SNPs in *MMP-2* and *MMP-3*.

		Control (n = 101)	PAP (n = 79)	*p* Value *
***MMP-2*/rs243865**				
Genotype	CC	65 (64.36)	55 (69.62)	
	CT	34 (33.66)	21 (26.58)	*p* = 0.343 OR = 0.73 CI = 0.38 to 1.40
	TT	2 (1.98)	3 (3.80)	*p* = 0.534 OR = 1.77 CI = 0.29 to 10.99
	CT + TT	36(37.62)	24 (30.38)	*p* = 0.358 OR = 0.75 CI = 0.40 to 1.39
Allele	C	164 (81.19)	131 (82.91)	
	T	38 (18.82)	27 (17.09)	*p* = 0.673 OR = 0.89 CI = 0.52 to 1.53
***MMP-2*/rs2285053**				
Genotype	CC	11 (10.89)	15 (18.99)	
	CT	49 (48.52)	33 (41.77)	*p* = 0.119 OR = 0.49 CI = 0.20 to 1.21
	TT	41 (40.59)	31 (39.24)	*p* = 0.200 OR = 0.55 CI = 0.22 to 1.37
	CT + TT	90 (89.11)	64 (81.01)	*p* = 0.125 OR = 0.52 CI = 0.22 to 1.21
Allele	C	71 (35.15)	63 (39.87)	
	T	131 (64.85)	95 (60.13)	*p* = 0.357 OR = 0.82 CI = 0.53 to 1.26
***MMP-2*/rs2287074**				
Genotype	GG	41 (40.59)	35 (44.30)	
	GA	49 (48.52)	35 (44.30)	*p* = 0.576 OR = 0.84 CI = 0.45 to 1.56
	AA	11 (10.89)	9 (11.40)	*p* = 0.933 OR = 0.96 CI = 0.36 to 2.58
	GA + AA	60 (59.41)	44 (55.70)	*p* = 0.617 OR = 0.86 CI = 0.47 to 1.56
Allele	G	131 (64.85)	105 (66.46)	
	A	71 (35.15)	53 (33.54)	*p* = 0.751 OR = 0.93 CI = 0.60 to 1.44
***MMP-3*/rs679620**				
Genotype	GG	27 (26.73)	20 (25.32)	
	GA	54 (53.47)	42 (53.16)	*p* = 0.892 OR = 1.05 CI = 0.52 to 2.12
	AA	20 (19.80)	17 (21.52)	*p* = 0.756 OR = 1.15 CI = 0.48 to 2.73
	GA + AA	74 (73.27)	59 (74.68)	*p* = 0.830 OR = 1.08 CI = 0.55 to 2.11
Allele	G	108 (53.46)	82 (51.90)	
	A	94 (46.54)	76 (48.10)	*p* = 0.768 OR = 1.06 CI = 0.70 to 1.62
***MMP-3*/rs522616**				
Genotype	AA	68 (67.33)	39 (49.37)	
	AG	30 (29.70)	35 (44,30)	*p* = **0.025** OR = 2.03 CI = 1.09 to 3.81
	GG	3 (2.97)	5 (6.33)	*p* = 0.144 OR = 2.91 CI = 0.66 to 12.82
	AG + GG	33(32.67)	40 (50.63)	*p* = **0.015** OR = 2.11 CI = 1.15 to 3.87
Allele	A	166 (82.18)	113 (71.52)	
	G	36 (17.82)	45 (28.48)	*p* = **0.016** OR = 1.84 CI = 1.11 to 3.02

* chi-square test; OR: odds ratio; CI: confidence interval. In bold, *p* < 0.05 = statistically significant.

## Data Availability

The original contributions presented in this study are included in the article. Further inquiries can be directed to the corresponding author.
